# Patient Preferences for Biologicals in Psoriasis: Top Priority of Safety for Cardiovascular Patients

**DOI:** 10.1371/journal.pone.0144335

**Published:** 2015-12-03

**Authors:** Marthe-Lisa Schaarschmidt, Christian Kromer, Raphael Herr, Astrid Schmieder, Diana Sonntag, Sergij Goerdt, Wiebke K. Peitsch

**Affiliations:** 1 Department of Dermatology, University Medical Center Mannheim, Heidelberg University, Mannheim, Germany; 2 Mannheim Institute of Public Health, Social and Preventive Medicine, Medical Faculty Mannheim, Heidelberg University, Mannheim, Germany; Kermanshah University of Medical Sciences, ISLAMIC REPUBLIC OF IRAN

## Abstract

Patients with psoriasis are often affected by comorbidities, which largely influence treatment decisions. Here we performed conjoint analysis to assess the impact of comorbidities on preferences of patients with moderate-to-severe psoriasis for outcome (probability of 50% and 90% improvement, time until response, sustainability of success, probability of mild and severe adverse events (AE), probability of ACR 20 response) and process attributes (treatment location, frequency, duration and delivery method) of biologicals. The influence of comorbidities on Relative Importance Scores (RIS) was determined with analysis of variance and multivariate regression. Among the 200 participants completing the study, 22.5% suffered from psoriatic arthritis, 31.5% from arterial hypertension, 15% from cardiovascular disease (myocardial infarction, stroke, coronary artery disease, and/or arterial occlusive disease), 14.5% from diabetes, 11% from hyperlipidemia, 26% from chronic bronchitis or asthma and 12.5% from depression. Participants with psoriatic arthritis attached greater importance to ACR 20 response (RIS = 10.3 vs. 5.0, p<0.001; β = 0.278, p<0.001) and sustainability (RIS = 5.8 vs. 5.0, p = 0.032) but less value to time until response (RIS = 3.4 vs. 4.8, p = 0.045) than those without arthritis. Participants with arterial hypertension were particularly interested in a low risk of mild AE (RIS 9.7 vs. 12.1; p = 0.033) and a short treatment duration (RIS = 8.0 vs. 9.6, p = 0.002). Those with cardiovascular disease worried more about mild AE (RIS = 12.8 vs. 10, p = 0.027; β = 0.170, p = 0.027) and severe AE (RIS = 23.2 vs. 16.2, p = 0.001; β = 0.203, p = 0.007) but cared less about time until response (β = -0.189, p = 0.013), treatment location (β = -0.153, p = 0.049), frequency (β = -0.20, p = 0.008) and delivery method (β = -0.175, p = 0.023) than others. Patients’ concerns should be addressed in-depth when prescribing biologicals to comorbid patients, keeping in mind that TNF antagonists may favourably influence cardiovascular risk.

## Introduction

Psoriasis is a chronic immune-mediated disease primarily affecting the skin but associated with a systemic inflammatory constellation. Approximately 20–30% of patients with psoriasis develop psoriatic arthritis. Furthermore psoriasis is associated with a wide range of metabolic and cardiovascular comorbidities [1,2]. On the one hand, these comorbidities may be provoked by unhealthy lifestyle behaviour such as increased food consumption, smoking and alcohol intake [3]. On the other hand, systemic inflammation in psoriasis is a key factor triggering cardiovascular risk. In a concept designated psoriatic cascade, it has been proposed that the chronic systemic inflammation leads to endothelial dysfunction and insulin resistance which trigger atherosclerosis [4]. Moreover, psoriasis, metabolic and cardiovascular comorbidities share certain genetic risk factors [5]. The relative risk of cardiovascular events is highest for young patients with psoriasis and increases with rising severity of the psoriasis [3]. Severe psoriasis was reported to confer an additional 6% risk to the 10-year rate of major adverse cardiovascular events [6].

Patients with psoriasis are also at increased risk of depression and suicide [7,8]. On the one hand, clinical symptoms of psoriasis, impaired disease-related quality of life, shame, social stigmatization and professional discrimination can result in severe psychological distress. On the other, depression may lead to a vicious circle of frustration, discouragement, non-adherence to treatment and thereby worsening of the psoriasis. Interestingly, psoriasis and depression have an overlapping profile of pro-inflammatory cytokines [9]. However, not only the psoriasis itself but also metabolic and cardiovascular comorbidities can contribute to the development of depressive symptoms [7].

A wide range of therapeutic options is available for psoriasis, including topical therapy, phototherapy, traditional systemic antipsoriatic medications and biologicals [10]. In Germany the TNF antagonists adalimumab, etanercept and infliximab and the interleukin 12/23 antagonist ustekinumab have been at hand for treatment of moderate-to-severe psoriasis and psoriatic arthritis during the last years. All of these medications are highly effective with a favourable benefit-risk profile, but they possess certain differences in efficacy, rapidity of action and sustainability. Fastest onset of action and highest chances of achieving reduction of the Psoriasis Area and Severity Index (PASI) have been reported for infliximab, followed by ustekinumab and adalimumab [11,12]. American College of Rheumatology (ACR) response rates for psoriatic arthritis are somewhat higher for TNF antagonists than for ustekinumab [13,14]. Moreover, the treatment process of each biological, i.e., the mode and frequency of application, is different. TNF antagonists were demonstrated to beneficially influence cardiovascular risk whereas the impact of ustekinumab on cardiovascular risk is less clear [15,16].

Despite various therapeutic options, treatment dissatisfaction and non-adherence are common among patients with psoriasis [17,18]. However, treatment satisfaction with biologicals is considerably higher than with other treatment modalities [19,20]. Higher levels of treatment satisfaction, along with higher likelihood of adherence and improved outcome, can be reached by increased incorporation of patient preferences into treatment decisions [21]. A proper approach for elicitation of preferences is conjoint analysis (CA, discrete choice experiments), a method originating from marketing research that has gained increasing popularity in the health care sector. This method provides the advantage of realistically reflecting decision processes in daily clinical practice [22,23].

In our previous study we used CA to assess general treatment preferences of patients with psoriasis for biologicals and demonstrated highest utilities for safety and efficacy [24]. In addition, when investigating patient preferences for all treatment modalities available for psoriasis (i.e., topical therapy, phototherapy, traditional systemic antipsoriatic medication and biologicals), we noted high impact of comorbidities on preferences [25]. The aim of the present study was to assess the impact of comorbidities on patient preferences for treatment of psoriasis with biologicals by using CA. We show that preferences vary significantly dependent on presence or absence of psoriatic arthritis, cardiovascular disease and diabetes.

## Materials and Methods

### Study participants

Patients with moderate-to-severe psoriasis visiting outpatient clinics at the Department of Dermatology of the University Medical Center Mannheim, Germany were asked to participate. Inclusion criteria were age ≥18 years and moderate-to-severe psoriasis according to the criteria of the Committee for Medicinal Products for Human Use (CHMP; for details, see [23]). Exclusion criteria were inability to complete the survey due to difficulties with German language or inability to understand CA exercises (i.e., failing to answer exercises with unambiguous scenarios presented for control). Both patients seeking consultation for the first time and patients coming for follow-up visits were included. The study was performed according to the principles of the Declaration of Helsinki and approved by the Ethics Committee of the Medical Faculty Mannheim (Ethics Approval 2009-329E-MA, 22 October 2009; Amendment 27 September 2012).

### Data collection

Participants who had provided written informed consent were asked to complete a computerized survey before clinical consultation. The survey contained information on sociodemographic characteristics (age and gender), Dermatology Life Quality Index (DLQI), comorbidities, and smoking habits (current smoker (defined as currently smoking ≥1 cigarette per day), former smoker, or non-smoker). Comorbidities were assessed by choosing out of a list of options including psoriatic arthritis, diabetes, depression, allergies, arterial hypertension, cardiovascular disease (myocardial infarction, stroke, coronary artery disease, and/or arterial occlusive disease), chronic bronchitis or asthma, hyperlipidemia, liver disease and neoplasia. In addition, participants were asked to indicate other comorbidities as free text. If patients reported arthralgia and/or suspected psoriatic arthritis, Classification for Psoriatic Arthritis (CASPAR) criteria were applied to verify the diagnosis [26]. The PASI was determined by two of the investigators (M.-L.S. and C.K.).

Respondents’ preferences for treatment of psoriasis with biologicals were elicited by CA exercises, generated basically as described [27,28]. Seven key outcome attributes (probability of 50% improvement, probability of 90% improvement, time until response, sustainability of success, probability of mild AE, probability of severe AE, and probability of ACR 20 response) and four process attributes (treatment location, frequency, duration, and delivery method) were identified and decomposed into four levels describing adalimumab, etanercept, infliximab and ustekinumab as realistically as possible (S1 Table; for procedures and references regarding identification of attribute levels and for further details on generation of discrete choice scenarios see [24]). The probability of 50% improvement was depicted as the chance of reduction of the psoriasis by half, the probability of 90% improvement as the chance of almost complete clearance. Mild infections, injection-site reactions, headache, gastrointestinal symptoms and mild temporary change of laboratory parameters were indicated as examples of mild AE, tuberculosis, other severe infections, severe intolerance reactions and autoimmune diseases as examples of severe AE (for examples of discrete choice scenarios presented to the participants see [24]). Participants were confronted with hypothetical treatment scenarios generated by commercially available CA software (http://www.sawtoothsoftware.com) and repetitively asked to choose their individually preferred option out of pair-wise presented treatment scenarios. Preferences were measured by the software as described previously [24]. Subgroup analyses according to comorbidities and smoking status was performed with one-way ANOVAs (analysis of variance), using SPSS software. Brown-Forsythe tests were applied if the assumption of homogeneity of variances was not fulfilled.

### Regression analysis

Multivariate linear regression analysis was performed to estimate independent associations of gender, age, PASI, DLQI, psoriatic arthritis, cardiovascular disease, diabetes and depression with the dependent variable RIS. Arterial hypertension was not integrated into the regression models because of multicollinearity with cardiovascular disease. Asthma and chronic bronchitis were not considered because these comorbidities are not directly related to psoriasis. Hyperlipidemia, allergies and the smoking status were not included because bivariate statistics showed no significant association with RIS. A standardized regression coefficient β was assigned to each independent variable, indicating the amount of change in RIS when varying one of the variables while holding the others constant. P-values ≤0.05 were considered significant.

## Results

Out of 239 patients with moderate-to-severe psoriasis approached for study participation, 210 provided written informed consent. Ten participants had to be excluded due to problems with German language (n = 9) or inability to understand the CA exercises (n = 1). Two hundred participants completed the study survey.

Most of the participants were male (57.5%), and the mean age of the study population was 50.8 years (Table 1). The vast majority (95.5%) came for a follow-up visit, and 99% obtained antipsoriatic treatment (76.5% topical therapy, 10% phototherapy, 37.5% traditional systemic medications and 43.5% biologicals). Therefore the mean PASI was only 3.4 (range: 0–26.7). The mean DLQI was 6.2 (range: 0–30), reflecting moderate impairment of disease-related quality of life. 22.5% of the participants suffered from psoriatic arthritis, 31.5% from arterial hypertension, 15% from cardiovascular disease (myocardial infarction, stroke, coronary artery disease, and/or arterial occlusive disease), 14.5% from diabetes and 11% from hyperlipidemia. 26% reported chronic bronchitis or asthma and 25.5% allergies. Liver disease was documented in 2.5%, inflammatory bowel disease in 1%, rheumatoid arthritis in 2% and a history of neoplasia in 4% of the participants. The self-reported prevalence of depression was 12.5%, the prevalence of smoking 37.5% (Table 1).

**Table 1:** Characteristics of the study cohort.

Category	N (%)^1^
**Gender**	
Female	85 (42.5)
Male	115 (57.5)
**Age (years)**	
Mean (SD)	50.8 (14.1)
Median (min-max; IQR)	51 (18–84; 17.8)
**PASI**	
Mean (SD)	3.4 (4.1)
Median (min-max; IQR)	2 (0–26.7; 4.4)
**DLQI**	
Mean (SD)	6.2 (7.1)
Median (min-max; IQR)	4 (0–30; 9)
**Comorbidities**	
Psoriatic arthritis	45 (22.5)
Arterial hypertension	63 (31.5)
Cardiovascular disease	30 (15)
Diabetes mellitus	29 (14.5)
Hyperlipidemia	22 (11)
Allergies	51 (25.5
Chronic bronchitis / asthma	52 (56)
Depression	25 (12.5)
Liver disease	5 (2.5)
Inflammatory bowel disease	2 (1)
Rheumatoid arthritis	4 (2)
Neoplasia	8 (4)
Other comorbidities^2^	27 (13.5)
**Smoking habits**	
Current smoker	75 (37.5)
Former smoker	69 (34.5)
Never smoked	56 (28.0)

^1^ Mean (SD) and median (min-max; IQR) are recorded for age, PASI and DLQI. N (%) are indicated for all other variables.

^2^ Other comorbidities comprised hypo- or hyperthyroidism (n = 6), arthrosis (n = 5), latent tuberculosis (n = 2), osteoporosis (n = 2), tachycardia (n = 1), hyperuricemia (n = 1), chronic kidney disease (n = 1), peptic ulcer (n = 1), chronic tonsillitis (n = 1), borreliosis (n = 1), lupus erythematosus (n = 1), fibromyalgia (n = 1), coagulopathy (n = 1), multiple sclerosis (n = 1), brain stimulator (n = 1), epilepsy (n = 1), tinnitus (n = 1), sudden hearing loss (n = 1), glaucoma (n = 1), and chronic hand eczema (n = 1). Some participants reported more than one of these comorbidities.

DLQI: Dermatology Life Quality Index; IQR: interquartile range; max: maximum; min: minimum; N: number; PASI: Psoriasis Area and Severity Index; SD: standard deviation.

### Impact of comorbidities on preferences

Treatment preferences averaged across the whole study cohort were previously reported [24]. Briefly, participants attached greatest value to the risk of severe AE (RIS = 17.3), followed by probability of 90% improvement (RIS = 14.0) and risk of mild AE (RIS = 10.5). Time until response (RIS = 4.5), sustainability of success (RIS = 5.2) and probability of ACR 20 response (RIS = 6.2) were considered least important. All process attributes reached intermediate RIS, ranging from 8.2 to 8.8.

**Fig 1:**
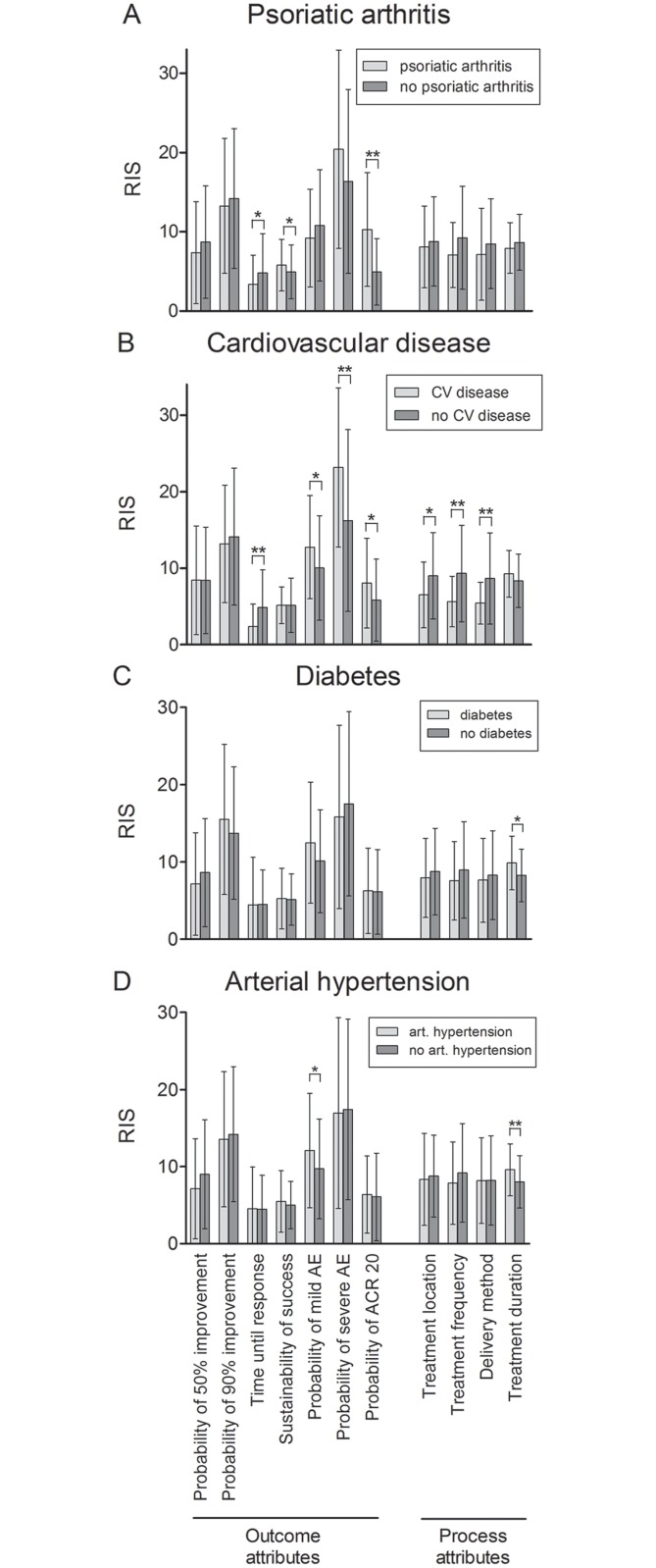
Impact of comorbidities on preferences for outcome and process attributes of biologicals. (A) Participants with psoriatic arthritis attached higher value to probability of ACR 20 response and sustainability but less importance to time until response. (B) Respondents with cardiovascular disease prioritized avoidance of mild and severe AE as well as ACR 20 response. Time until response, treatment location, frequency and delivery method were less relevant for them than for other participants. (C) Participants with diabetes were particularly interested in short treatment duration. (D) Those with arterial hypertension attached special importance to a low risk of mild AE and a short treatment duration. Differences in RIS were tested for significance with one-way ANOVA or Brown-Forsythe tests. Bars: Means with standard deviations. AE: adverse events; art.: arterial; CV disease: cardiovascular disease; RIS: Relative Importance Scores. * p≤0.05, ** p≤0.01.

Participants suffering from psoriatic arthritis placed greater importance on the probability of ACR 20 response (RIS = 10.3 vs. 5.0, p<0.001), sustainability of the therapeutic success (RIS = 5.8 vs. 5.0, p = 0.032) and by trend on probability of severe AE (RIS = 20.4 vs. 16.4, p = 0.052) than participants without arthritis (Fig 1A). By contrast, time until response was less relevant from their perspective (RIS = 3.4 vs. 4.8, p = 0.045). Multivariate regression analysis controlling for gender, age, PASI, DLQI and other comorbidities (cardiovascular disease, diabetes and depression) confirmed significantly higher importance of ACR 20 response in case of psoriatic arthritis (β = 0.278, p<0.001, Table 2).

**Table 2:** Multiple linear regression models demonstrating impact of comorbidities on outcome attributes.

Outcome attributes														
Characteristic	Probability of 50% improvement		Probability of 90% improvement		Time until response		Sustainability of success		Probability of mild AE		Probability of severe AE		Probability of ACR 20 response	
	β	p	β	p	β	p	β	p	β	p	β	p	β	p
**PsA**	-.041	.569	.023	.745	-.109	.131	.128	.084	-.119	.105	.089	.217	**.278**	**< .001**
**CV disease**	.037	.629	-.025	.741	**-.189**	**.013**	.004	.960	**.170**	**.027**	**.203**	**.007**	.115	.121
**Diabetes**	.008	.917	.134	.069	-.017	.812	-.021	.775	.053	.478	-.084	.252	-.037	.610
**Depression**	-.024	.741	.083	.244	.057	.421	-.109	.140	.003	.970	-.126	.079	.005	.945

The Relative Importance Score (RIS) was defined as dependent variable. Gender, age, PASI, DLQI, psoriatic arthritis (PsA), cardiovascular (CV) disease, diabetes and depression were used as predictors. β represents the standardized regression coefficient. A positive β indicates a higher importance of the attribute compared to the reference group. The reference group for each disease comprised participants who did not suffer from this condition. Significant findings are highlighted in bold.

When participants were stratified according to presence or absence of cardiovascular comorbidities, prevalence of psoriatic arthritis was somewhat higher in the subgroup with cardiovascular disease (33.3% vs. 20.6%, p = 0.123). Participants with cardiovascular comorbidities worried more about mild AE (RIS = 12.8 vs. 10, p = 0.027, Fig 1B; β = 0.170, p = 0.027 in multivariate regression models, Table 2), severe AE (RIS = 23.2 vs. 16.2, p = 0.001; β = 0.203, p = 0.007) and probability of ACR 20 response (RIS = 8.1 vs. 5.8, p = 0.029; insignificant in multivariate regression models) than others. However, they cared less about time until response (RIS = 2.4 vs. 4.9, p = 0.001; β = -0.189, p = 0.013, Table 2) and assigned less importance to most process attributes (treatment location: RIS = 9.0 vs. 6.5, p = 0.02; treatment frequency: RIS = 5.6 vs. 9.3, p<0.001, delivery method: RIS = 5.4 vs. 8.7, p = 0.001; Fig 1B). When adjusting for socio-demographic factors, PASI, DLQI and other comorbidities, the impact of cardiovascular disease on all of these process attributes remained significant (for standardized β-values and corresponding p-values see Table 3).

**Table 3:** Multiple linear regression models showing the influence of comorbidities on process attributes.

Process attributes								
Characteristic	Treatment location		Treatment frequency		Delivery method		Treatment duration	
	β	p	β	p	β	p	β	p
**PsA**	-.023	.757	-.112	.118	-.113	.124	-.083	.249
**CV disease**	**-.153**	**.049**	**-.201**	**.008**	**-.175**	**.023**	.105	.168
**Diabetes**	-.012	.870	-.051	.484	-.018	.814	.092	.213
**Depression**	.080	.273	-.009	.900	.072	.319	-.044	.540

For explanations and abbreviations, see Table 2.

Subgroup analysis with respect to diabetes revealed that participants with diabetes were more interested in treatment duration than others (RIS = 9.9 vs. 8.3, p = 0.023, Fig 1C). However, this finding was not confirmed in multivariate analyses.

Partcipants with arterial hypertension were particularly interested in a low risk of mild AE (RIS 9.7 vs. 12.1; p = 0.033) and in a short treatment duration (RIS = 8.0 vs. 9.6, p = 0.002; Fig 1D). Similarly, participants with chronic bronchitis or asthma attached special importance to avoiding mild AE (RIS = 10.1 vs. 13.7, p = 0.034). Hyperlipidemia, allergies and the smoking status had no significant impact on preferences (data not shown). Furthermore, preferences of participants with or without self-reported depression did not differ significantly from each other, neither in bivariate analyses (data not shown) nor in multivariate regression models (Tables 2 and 3). Subgroup analyses with respect to other comorbidities were precluded by the small sample sizes.

## Discussion

The burden of comorbidities, most importantly, psoriatic arthritis, cardiovascular disease, diabetes and depression, may exert an enormous incremental impact on quality of life and work productivity of patients with psoriasis [27]. These comorbidities have great influence on treatment decisions, including decisions in favour of or against prescription of biologicals. For example, TNF antagonists are regarded as biologicals of first choice in patients with psoriatic arthritis [28]. However, they are contraindicated in patients with high-grade cardiac insufficiency [15,29]. Here we show that comorbidities strongly influence patients’ preferences for attributes of biologicals.

Participants with psoriatic arthritis were more interested in improvement of arthralgia than others, as expected. In addition, they were willing to trade rapidity of success in favour of increased sustainability. Psoriatic arthritis leads to more severe impairment in physical functions and daily work and leisure activities than mere cutaneous psoriasis [30]. Therefore, identification of a highly effective and sustainable systemic antipsoriatic medication is even more compulsory for patients with psoriatic arthritis.

Participants suffering from cardiovascular disease were particularly concerned about AE and willing to accept slower onset of action and a less convenient treatment process in order to minimize the risk of AE. These patients may have a special interest in avoiding additional risk as they might have experienced life threatening events such as angina pectoris, myocardial infarction and stroke. Furthermore, they are likely to receive multi-drug comedication [31] and therefore more prone to AE due to drug interactions. However, systemic inflammation in psoriasis is an important trigger of cardiovascular risk and efficient control of this inflammation is crucial for cardiovascular risk reduction [4,15,32]. TNF antagonists were convincingly demonstrated to reduce the risk of cardiovascular events both in rheumatoid arthritis [33] and in psoriasis [15,16,32,34]. Patients with psoriasis receiving TNF antagonists or methotrexate have lower cardiovascular event rates than patients treated with other medications [16,32]. Use of TNF antagonists is associated with a reduced incidence of myocardial infarction as compared to topical treatment [34]. Moreover, there is evidence that TNF antagonists have a beneficial effect on preventing the progression of subclinical atherosclerosis and arterial stiffness in patients with psoriasis and inflammatory arthritis [35,36,37]. According to a recent systematic review based on 23 studies, TNF-α inhibitors were convincingly shown to prevent or even reverse the progression of intima media thickness in patients with rheumatoid arthritis, ankylosing spondylitis and psoriatic arthritis who respond to treatment [37]. In addition, several studies pointed to a reduction of the pulse wave velocity as indicator of arterial stiffness upon treatment with TNF antagonists [37]. Last but not least, TNF antagonists improve biomarkers of inflammation such as C-reactive protein [38], which are independent risk factors of atherosclerosis.

The impact of ustekinumab on cardiovascular risk is less clear. As another interleukin 12/23 antagonist, briakinumab, was withdrawn from further development in part due to concerns about cardiovascular safety, discussion has also emerged on cardiovascular safety of ustekinumab. Associations between the two interleukin 12/23 antagonists and major adverse cardiovascular events (MACEs) were examined in two meta-analyses of randomized controlled trials [39,40]. Even if the same trials and the same number of MACEs were included, these meta-analyses came to different conclusions. According to Ryan et al. the risk of MACEs was not significantly elevated with use of interleukin 12/23 antagonists [39]. Tzellos and colleagues used another statistical methodology and found a more than fourfold increased odds ratio for MACEs with interleukin 12/23 antagonists ([40]; for discussion of these discrepancies, see [41]). An integrated analysis of data from phase II and III studies with ustekinumab alone suggested that ustekinumab use was associated with a decrease in MACEs compared to the general US population or to psoriasis patients in Great Britain [42]. In addition, 5-year safety data for ustekinumab did not show an increased risk of MACEs compared to the general population [43]. According to a Danish nationwide cohort study, the risk of composite cardiovascular endpoints was significantly decreased upon treatment with TNF antagonists, whereas patients receiving ustekinumab had a similar risk as the reference group using other therapies [16]. However, discussion on cardiovascular safety of ustekinumab has still not been fully resolved [44,45].

Our finding that patients with cardiovascular comorbidities are particularly worried about AE is in line with results from our previous study in which we compared preferences of comorbid patients for all kinds of antipsoriatic treatments without addressing attributes of specific medications [25]. Moreover, it is well compatible with the observation that participants with other chronic diseases such as arterial hypertension and chronic bronchitis or asthma are especially concerned about safety. Suffering from a chronic disease which probably requires long-term treatment and life-long surveillance is likely to sensitize patients for safety aspects. Our results highlight the importance of carefully discussing safety issues with patients affected by such chronic and potentially threatening diseases and explicitly addressing their concerns about AE. It is essential to explain that reduction of systemic inflammation can beneficially influence cardiovascular risk, a fact most patients are unaware of.

Participants with diabetes as well as participants with arterial hypertension placed greater value on treatment duration than others. Management of diabetes can be quite complex and time-consuming. Therefore it is comprehendible that patients suffering both from psoriasis and from diabetes prefer a time-saving treatment. Interestingly, there is increasing evidence that TNF antagonists may improve insulin resistance in patients with psoriasis and rheumatoid arthritis [46,47,48]. A recent study on 29 non-diabetic individuals with moderate-to-severe psoriasis disclosed a statistically significant improvement of insulin sensitivity after 6 months of adalimumab therapy [48]. Significant improvement of erythrocyte sedimentation rate, ultrasensitive CRP, BSA, PASI, Nail Psoriasis Severity Index, Physician Global Assessment and psoriatic arthritis screening and evaluation questionnaire was also observed at that time [48]. Similarly, amelioration of insulin sensitivity was noted in non-diabetic patients with rheumatoid arthritis receiving TNF-α antagonist therapy [47]. A beneficial effect has also been observed on retinol-binding protein-4, an adipokine considered as an emerging cardiometabolic risk factor that is increased in patients with moderate-to-severe psoriasis [49]. Moreover, psoriasis patients treated with TNF antagonists have a lower risk of new-onset diabetes than patients on non-biological disease-modifying antipsoriatic drugs [50].

When we compared preferences for all antipsoriatic treatment modalities, we found that patients with depression were particularly interested in a time-saving therapy with low individual costs [25]. The CA experiments presented here did not contain a cost attribute because treatment costs for biologicals are covered by health insurance in Germany if their prescription is accordant to licence. Depression did not significantly impact preferences for treatment with biologicals, possibly because their application is more convenient and time-saving than topical therapy and phototherapy. Both TNF antagonists and ustekinumab can substantially improve symptoms of depression and health-related quality of life in patients with psoriasis [51,52].

A limitation of our study is that data on the prevalence of comorbidities were based on patients' self-reported medical history. This way of data collection may lead to underestimation of the frequency of comorbidities due to reporting bias. However, patients are likely to reliably remember and report diabetes and severe cardiovascular diseases such as myocardial infarction, stroke and coronary artery disease. Diagnosis of psoriatic arthritis was either previously confirmed by a physician or verified by us with CASPAR criteria which have >98% specificity [26]. Self-reporting of depression may result both in underestimation of its prevalence due to social desirability bias and in overestimation if patients interpret transient slight depressive moods as depression. In clinical practice, this comorbidity is frequently underdiagnosed [53].

Moreover it has to be emphasized that in addition to the comorbidities considered in detail in this study, psoriasis is associated with other diseases influencing cardiovascular risk, in particular, non-alcoholic fatty liver disease, inflammatory bowel disease, rheumatoid arthritis and other autoimmune diseases [54,55]. The number of participants with liver disease, inflammatory bowel disease and rheumatoid arthritis in our cohort was rather small, precluding subgroup analyses with respect to these comorbidities. It is well conceivable that non-alcoholic fatty liver disease was underdiagnosed and that its true prevalence was underestimated, because information on this comorbidity was merely based on patient reports instead of being verified by laboratory tests and ultrasound.

A further limitation is that only patients with moderate-to-severe psoriasis treated at a German University Hospital were included, i.e., relatively severely affected patients with long disease duration (mean: 19.9 years) and extensive treatment experience. Clearly, our findings will have to be confirmed in larger cohorts and in a multi-centric setting.

The mean PASI of the study collective was low, because the vast majority of respondents received antipsoriatic therapy at the time of data collection. However, all participants suffered from moderate-to-severe psoriasis according to CHMP criteria, and all were potential candidates for the biologicals. Forty-six percent of the participants had experience with biologicals, which may influence preferences.

In conclusion, we show that patient preferences for biologicals are considerably influenced by their comorbidities. Patients with cardiovascular disease and arterial hypertension are particularly concerned about safety, even if TNF antagonists favourably influence cardiovascular risk. Clearly, treatment decisions cannot merely depend on patient preferences but have to be based on medical conditions, and physicians’ recommendations may deviate from patient preferences because of medical reasons [56]. Nevertheless it is essential to address patients’ needs and concerns and to discuss individual benefits and risks of comorbid patients in-depth in order to ensure a trustful physician-patient relationship and optimize adherence and outcome.

## Supporting Information

S1 Table Outcome and process attributes and attribute levels. ^1^ Probability of loss of response within one year. ^2^ per treatment session. AE: adverse events.
(DOC)
