# Supplementary material for: Patient Preferences for Biologicals in Psoriasis: Top Priority of Safety for Cardiovascular Patients
Source: PLoS One. 2015 Dec 3;10(12):e0144335. doi: 10.1371/journal.pone.0144335 (PMC4669171; doi:10.1371/journal.pone.0144335)
Supplement: S1 Table — 1 Probability of loss of response within one year. 2 per treatment session. AE: adverse events. (DOC) [file pone.0144335.s001.doc]

S1 Table. Outcome and process attributes and attribute levels.

| **Outcome attribute** | **Level** |
| --- | --- |
| Probability of 50% improvement | 90-95%  85-90%  80-85%  70-80% |
| Probability of 90% improvement | 50-60%  40-50%  30-40%  20-30% |
| Time until response | 2 weeks  4 weeks  8 weeks  12 weeks |
| Sustainability of therapeutic success^1^ | 20%  15%  10%  5% |
| Probability of mild AE | 50-70%  30-50%  10-30%  <10% |
| Probability of serious AE | 5-10%  2-5%  1-2%  <1% |
| **Process attribute** | **Level** |
| Treatment location | at home  at a general practitioner’s office  at a dermatologist’s office  as an outpatient in a hospital |
| Treatment frequency | once to twice per week  every two weeks  every 4-8 weeks  every 12 weeks |
| Delivery method | syringes into the subcutaneous fatty tissue administered by the patient  syringes into the subcutaneous fatty tissue administered by medically trained persons  injections into the subcutaneous fatty tissue with a pen administered by the patient  infusions administered by a doctor |
| Treatment duration^2^ | 5 minutes  15-30 minutes  1 hour  2-3 hours |

^1^ Probability of loss of response within one year

^2^ per treatment session

AE: adverse events
